# The Morra Game as a Naturalistic Test Bed for Investigating Automatic and Voluntary Processes in Random Sequence Generation

**DOI:** 10.3389/fpsyg.2020.551126

**Published:** 2020-09-24

**Authors:** Franco Delogu, Madison Barnewold, Carla Meloni, Enrico Toffalini, Antonello Zizi, Rachele Fanari

**Affiliations:** ^1^Department of Humanities, Social Sciences and Communication, Lawrence Technological University, Southfield, MI, United States; ^2^Department of Pedagogy, Psychology, Philosophy, University of Cagliari, Cagliari, Italy; ^3^Department of General Psychology, University of Padua, Padua, Italy

**Keywords:** random number generation, central executive, automatic processing, proceduralization, hand game, dual processing theory, course-based research experience

## Abstract

Morra is a 3,000-years-old hand game of prediction and numbers. The two players reveal their hand simultaneously, presenting a number of fingers between 1 and 5, while calling out a number between 2 and 10. Any player who successfully guesses the summation of fingers revealed by both players scores a point. While the game is extremely fast-paced, making it very difficult for players to achieve a conscious control of their game strategies, expert players regularly outperform non-experts, possibly with strategies residing out of conscious control. In this study, we used Morra as a naturalistic setting to investigate the necessity of attentive control in generation of sequence of items and the ability to proceduralize random number generation, which are both a crucial defensive strategy in Morra and a well-known empirical procedure to test the central executive capacity within the working memory model. We recorded the sequence of numbers generated by expert players in a Morra tournament in Sardinia (Italy) and by undergraduate students enrolled in a course-based research experience (CRE) course at Lawrence Technological University in the United States. Number sequences generated by non-expert and expert players both while playing Morra and in a random number generation task (RNGT) were compared in terms of randomness scores. Results indicate that expert players of Morra largely outperformed non-experts in the randomness scores only within Morra games, whereas in RNGT the two groups were very similar. Importantly, survey data acquired after the games indicate that expert players have very poor conscious recall of their number generation strategies used during the Morra game. Our results indicate that the ability of generating random sequences can be proceduralized and do not necessarily require attentive control. Results are discussed in the framework of the dual processing theory and its automatic-parallel-fast vs. controlled-sequential-slow polarities.

## Introduction

Morra is an ancient hand game still played nowadays. In its more diffuse version, two players extend one arm in front of the opponent to show a number of fingers, while simultaneously shouting a number from 2 to 10. The player who successfully guesses the total number of fingers shown by the two hands scores a point.

Morra is believed to have originated in ancient Egypt, as the first known iconographic trace of the game, dating back to about 4,000 years ago, has been found in the Beni Hasan tombs (Ebers, 1878; Falkener, 1892), a cemetery site of the Egypt middle kingdom. Vase paintings show that Morra was played in Greece since 400 B.C. (Perdrizet, 1898). The game was then popular during the Roman Empire (also cited by Cicero in his *De Officiis* 3.77) when through the Roman legions spread across the Mediterranean basin. Morra is still played in many countries nowadays in different variants. The Sardinian variant of Morra, which is the one we are going to analyze in this study, is typically played in teams, in which two teammates take turns in playing against the two opponents from another team. Whenever a point is scored, the player who scored keeps playing and the loser is substituted by his teammate. This procedure continues until one of the two teams cumulatively reaches the number of points necessary to win a game, typically sixteen, and the match is over.

When formalized in a pay-off matrix, like the ones often used in game theory, Morra outcomes can be described as reported in Table 1.

**TABLE 1:** Matrix of all winning combinations of player B while playing against player A.

Player B	Player A	1, 2	1, 3	1, 4	1, 5	1, 6	2, 3	2, 4	2, 5	2, 6	2, 7	3, 4	3, 5	3, 6	3, 7	3, 8	4, 5	4, 6	4, 7	4, 8	4, 9	5, 6	5, 7	5, 8	5, 9	5, 10
**1, 2**			1	1	1	1																				
**1, 3**								1	1	1	1															
**1, 4**													1	1	1	1										
**1, 5**																		1	1	1	1					
**1, 6**																							1	1	1	1
**2, 3**		1		1	1	1																				
**2, 4**							1		1	1	1															
**2, 5**												1		1	1	1										
**2, 6**																	1		1	1	1					
**2, 7**																						1		1	1	1
**3, 4**		1	1		1	1																				
**3, 5**							1	1		1	1															
**3, 6**												1	1		1	1										
**3, 7**																	1	1		1	1					
**3, 8**																						1	1		1	1
**4, 5**		1	1	1		1																				
**4, 6**							1	1	1		1															
**4, 7**												1	1	1		1										
**4, 8**																	1	1	1		1					
**4, 9**																						1	1	1		1
**5, 6**		1	1	1	1																					
**5, 7**							1	1	1	1																
**5, 8**												1	1	1	1											
**5, 9**																	1	1	1	1						
**5, 10**																						1	1	1	1	

From a cognitive point of view, Morra is a complex activity which involves, and possibly integrates, many perceptual, cognitive and motor processes. In fact, a Morra player needs to select two numbers, one to be shown to the opponent with the fingers and one to be spoken. In order to be successful, a player should select those numbers in a very careful way: the to-be-shown number should be difficult to predict and the to-be-said number should be selected in order to target the number the opponent will show. This requires memory of previously shown and said numbers by the player and by his/her opponent. All these operations are conducted in a very small amount of time (more than one round per second). Morra, like many other games requiring speeded responses (Recalde et al., 2018) is prone to mistakes. The most common error in beginner players is to play impossible hand-voice combinations, like the ones in the following examples: (1) Say “seven” while showing “one” (the opponent cannot show “six,” which would allow the player to score the point, because the hand has a maximum of five fingers to show). (2) Say and show the same number (the opponent will add at least “one,” making the sum automatically higher than the spoken number). (3) Show a hand number that is smaller than the voice number. These beginner errors are almost non-existent in expert players. A frequent mistake, which is not uncommon even in players with some level of expertise, is to repeatedly show the same hand number.

Morra analysis can provide a new approach to study the interaction between several cognitive functions in an ecological setting. As of today, no previous analysis of the cognition of Morra has been conducted. All knowledge we have about the cognitive mechanisms involved is anecdotal. Popular knowledge about the game seems to indicate that Morra is hardly a game of luck. An expert player, is able, in fact, to produce effective strategies which lead almost invariably to success against less skilled players. Moreover, anecdotal observation suggests that Morra might involve automatic and controlled cognitive processing.

Playing Morra is likely to involve multiple executive functions. Selective attention is necessary to focus on spoken numbers and the pattern of fingers in input. Response inhibition is likely to be involved in avoiding repetitive finger patterns. Working memory is required to retain numerical sequences over multiple rounds. Such high cognitive load is complicated by the necessity of a speeded response, which likely reduces accuracy and control (Heitz, 2014). It is precisely the simultaneous necessity of executive control and speed that makes the case of Morra particularly interesting. If we were to classify Morra according to the dual process theory (Stanovich and West, 2000), we would be challenged by the fact that the game appears to involve both *system 1* (associative, holistic, intuitive, automatic, relatively undemanding of cognitive capacity, and fast) and *system 2* (analytic, controlled, effortful, slow, ruled-based, and flexible). In fact Morra seems fast, automatic, intuitive, implicit, and undemanding of cognitive capacity, but at the same time also appears as rule-based, analytic, controlled, conscious, explicit, and effortful.

In Morra, we can distinguish between attack and defense strategies. We define the attempt to be unpredictable in the sequences of numbers shown with the hand as defensive strategies. For example, for player A playing against player B, not to show the same number too often, or not showing the same combination of two consequent numbers can be an effective defensive strategy. In fact, the unpredictability of player A can make harder for player B to guess the next number player A will show and to consequently produce the correct sum of the two hands. The best defensive strategy for a Morra player would be to produce random sequences. However, there is robust evidence that humans cannot produce random sequences of numbers and instead tend to produce highly predictable patterns in random number generation tasks (Brugger, 1997). The defensive strategies in Morra games offer a powerful test bed to analyze how humans deviate from ideal performance when trying to produce random sequences of numbers in a naturalistic setting. Defensive strategies can be analyzed through quantitative analyses of the hand sequences using the methods typical of the random number generation task (see for example Towse and Neil, 1998). On the other hand, understanding patterns in the numeric sequence of the opponent is an effective attack strategy. Player A can try to read patterns in the sequence of player B and for example target numbers that are frequently shown by player B or target a number that frequently follows another number in the sequence. Attack strategies can be analyzed through a quantitative analysis of sequences of spoken numbers.

In this paper we will focus on defensive strategies and in their theoretical value for the study of a variety of cognitive processes involved in the production of number sequences. In particular, the selection of numbers to show with the hand, from now on hand-numbers, seem to require mechanisms that are similar to the ones required in the random number generation task (RNGT). As reported in Baddeley et al. (1998) random generation of numbers, letters or other items, has been highly successful in disrupting executive behavior across a range of tasks from selecting an optimal next move in chess (Robbins et al., 1996) to the acquisition of artificial grammar (Dienes et al., 1991). As summarized by Towse and Neil (1998), experimental research on random generation indicates that response production relies on Executive Functions (Baddeley et al., 1986; Baddeley et al., 1998; Towse and Mclachlan, 1999; Miyake et al., 2000; Vandierendonck, 2000). Random generation has been extensively used in the past as a measure of the central executive component of working memory with behavioral methods (Baddeley et al., 1998; Schulz et al., 2012; Oomens et al., 2015) and in neuroimaging (Jahanshahi et al., 2000; Daniels et al., 2003), in healthy subjects and patients suffering from frontal lobe lesions (Spatt and Goldenberg, 1993), dementia (Brugger et al., 1996b), Parkinson’s disease (Brown et al., 1998), schizophrenia (Rosenberg et al., 1990).

The Random Number Generation Task (RNGT) is the most used variety of random generation tasks and requires participants to repeatedly select a number from a given range (typically the digits 1–6 or 1–9). Usually, participants produce sequences of 100 or 200 numbers with a frequency of one number per second, either saying the numbers aloud, or in alternative writing or typing the digits. Sequences produced in RNGT are usually analyzed for their level of randomness. There is long lasting evidence that humans are not good randomizers (Wagenaar, 1972 for a review of classic studies). Randomness is generally quantified in a negative sense, with measures of deviation from it. As there is not a single measure that can account for the level of randomness, we will focus on methods that are more closely related to the concerns that an ideal player of Morra would have when trying to produce unpredictable sequences of hand-numbers. Here is a list of randomness qualities that a Morra player should exhibit in order to optimize his/her defensive strategies. (1) For Morra players it is important not to display a specific hand-number too often. For example, showing “one” a disproportionate number of times would lead the opponent to target the too-often-displayed “ones.” An ideal strategy would be to display an equal amount of occurrences of all the five possible alternatives “one,” “two,” ‘three,” “four,” and “five.” More in general, when a random number generation is performed the performance is ideal when each response alternative is selected with equal frequency. (2) For Morra players it is also important not to play too often a so-called pair (a sequence of two hand-numbers in a row). Players should also be able to play as many possible pairs as they can. With five possible outcomes, the total number of diagrams (pairs of numbers) is 25. Players should try to include as many combinations of two numbers as they can. 3. Morra players should also try to variate their sequence as much as possible. They can achieve that by trying to cycle through all possible 5 responses within the shortest possible sequence. It is important to underline that each of the abovementioned strategies of randomization that are useful in defensive strategies in Morra find has been used by researchers to define strategies to achieve optimal randomness in RNGT (Towse and Neil, 1998).

Number generation in Morra and in RNGT is similar because both tasks require successful participants to generate sequence of random numbers. The versions of Morra and RNGT we used in this study use the same set of possible choices (numbers ranging from 1 to 5) and the same pace (about one number per second). However, it must be noted that random number generation in Morra and in the RNGT differ in many respects. While in Morra numbers are generated while playing against another person, to make the opponent guesses more difficult, in RNGT the participant is not competing against anyone. Also, while number generation in numbers in Morra is performed in association with other cognitive tasks, in RNGT it is performed in isolation. Finally, while in Morra the sequence of numbers is expressed through configurations of fingers, in RNGT they are verbalized.

The relationship between RNGT and attention has been explored by pairing a concurrent task to RNGT (see for example Baddeley et al., 1998). Interestingly, it seems that concurrent requirements (Towse and Valentine, 1997; Towse and Cheshire, 2007) and even the arrangement of the body position (Loetscher et al., 2008), impact either the randomness level or the magnitude of numbers generated during RNGT.

Within the theoretical debate about the relationship between RNGT and attention, Morra seems like a perfect test bed to verify, in an ecological setting, whether concurrent requirements impact the randomness of sequences. In fact, Morra is a complex multitasking activity that, together with the generation of a sequence of random numbers to display, requires players to generate a sequence of numbers to be said aloud, and an effort to keep in memory the sequences of numbers displayed by him/herself and the opponent. Such tasks can be conceptualized as concurrent tasks in RNGT, opening the field to a direct comparison of Morra and RNGT. Consequently, we can expect that random number generation in Morra, which is associated with several additional concurrent tasks, will produce stronger deviations from randomness than traditional RNGT. The Morra paradigm offers the possibility to verify if practice can reduce the interference effect of the concurrent cognitive load by investigating whether Morra expertise reduces the costs of multitasking in random number generation. Within this theoretical framework, it is therefore interesting to compare RNGT performances during Morra and during RNGT in expert and naive Morra players.

While previous studies used non-cooperative hand games, like Rock-Paper-Scissors, as a test bed to understand strategic interactions between humans (Wang et al., 2014), no previous studies used games in naturalistic setting to investigate random number generation. In this study we wanted to verify to which extent Morra expert players show higher proficiency than beginners in random number generation. We also wanted to investigate whether they can proceduralize the generation of random sequences of numbers. Operatively, we compared the performance of expert and beginners players of Morra while playing Morra and while performing RNGT. We expect Morra players to outperform beginners in Morra—but not in RNGT—in terms of randomness. More specifically, in terms of within-group comparisons, we expect to find metrics of randomness in expert players to be nearly equivalent when they play Morra or perform RNGT, whereas a large difference is expected to be found in beginners. Such findings would suggest that Morra players do not suffer the costs of multitasking while playing Morra because they are able to proceduralize the generation of random numbers. In contrast, we expect that non-experts will show largely impaired measures of randomness during Morra compared to RNGT. In our analysis, we compared metrics of randomness in experts and non-experts players obtained from number sequences produced during Morra games and during RNGT.

## Materials and Methods

### Participants

Eighteen participants took part in the study. The sample included 9 expert players of Morra (all male), mean age: 31.44, St. Dev. = 10.06), and 9 beginners, students at Lawrence Technological University (5 female, mean age: 20.11, St. Dev. = 1.19). As the experiment required the acquisition of data from a semi-naturalistic setting in an open-space (Morra tournament in a central square of a small town in Sardinia, Italy) and in a university setting (a course-based research experience in a research methods course in an American university), the sampling process presented some constraints in sample size, age and gender stratification. Specifically, the sample of experts was restricted to the number of players agreeing to participate in a 1-day-only tournament and be video recorded, and to the resulting amount of acceptable data. Concerning the choice of two groups with different mean age (expert players from Sardinia and college students from Lawrence Technological University), the choice of the sample was constrained by the specific experimental setting. The choice of the college student as the beginner group was dictated by the accessibility of the sample. In fact, the beginners’ group needed a training of several hours to be proficient enough in the game to be comparable to experts in terms of knowledge of the mechanics of the game. Also, they needed to be tested several times in a semester. Logistically, this training and testing was achieved by developing a course based research experience within a research methods course at LTU. Concerning gender, as Morra is a traditionally male-only game, it was not possible, even though we tried, to involve in the tournament the few female expert players we could find. As for the beginners ‘sample, since there is evidence for equivalent performance between female and male participants in RNGT (Brugger et al., 1996a) and no previous evidence about gender difference in Morra, we included all the students enrolled in the research methods class (female and male) in the beginner sample. While there are no data to support gender differences of equivalence, we do not see any theoretical reasons to predict that one gender would outperform the other after comparable training. The LTU non-expert group was trained to play at the same pace (about one play per second) as the Sardinian experts. All participants were allowed to choose their preferred hand to show the numbers.

The research was approved by the Institutional Review Board of Lawrence Technological University and by the Ethical Committee of the Department of Philosophy and Psychology of the University of Cagliari. Informed consents were signed by all participants before taking part on the experience.

### Procedure

#### Morra Tournament in Bitti, Sardinia, Italy

The Morra data from the expert players were acquired during a Morra tournament that was played in the town of Bitti (Sardinia, Italy) on August 5th, 2016. The tournament was organized with the specific purpose of recording Morra data and was sponsored and promoted by the *Comune di Bitti* (Bitti’s Town Hall), which hosted the event in the main public square of the town. Eight teams of two expert players were invited to take part in the event. The main criterion for inclusion in the sample was to be an expert player of Morra (regular participation in Morra tournament as a plus). Gender was not a criterion for selection, but we were not able to recruit any female players. The single-elimination tournament included 8 teams. Teams were randomly paired up with another team to play against. Each match included three games, the teams that won two out of three games proceeded to the next round, where they played against the winners of another match. The loser team was eliminated and no longer in the tournament. This elimination procedure continued until the final match, in which the winning team became the champion team. The winning team received a first place trophy and the runner up team that lost to them received a second place trophy. No monetary rewards were offered to any of the participating teams.

Beside the main tournament, a so-called “free-table” was also organized in which teams could sign-up for three-games matches against other teams.

Concerning the logistics of the event, five square tables were arranged in the public square at an average distance of 40 m from each other. Four of the tables were used for the tournament and one for the free-table event. All games of the tournament and the free-table were video recorded through video cameras placed on the top of each table, through telescopic booms, at an average distance of 1 m from the tabletop.

#### Morra Games at Lawrence Technological University, Michigan

The Morra data from the beginner players were acquired from the research method in the behavioral science class at Lawrence Technological University during the Fall 2017 semester. All students enrolled in the class participated as part of required activities for the class. At the beginning of the semester students played one-against-one games while in the second part of the semester students were paired into teams of two to play two-against-two games, following the traditional rules of Sardinian Morra. Each game that was played was video recorded with smartphone cameras. Registrations of Morra games, including both single-against-single and team games, were taken starting on September 6th, 2017 to November 29th, 2017. After each game, data about the sequence of spoken and hand numbers were tabulated into spreadsheets. Students had 12 weeks of experience, playing approximately twice a week. The data included in this study were taken from games in which students had Morra experience ranging from a few games (third week of classes) to about 30 games (at the end of the semester). Recordings were included in the final analysis only when a minimum amount of 57 numbers were played in the game. Each participant included in the final sample played at least four games including both one-against-one and team play games. Data from each participant included at least one team play game. In one-against-one games points were marked by the players themselves as they were earned and the first person to get 10 points was marked as the winner. During team games a referee kept score for both teams and the first team to reach 16 points was marked as the winner. Teams were randomly assigned to play against each other.

#### Random Number Generation Task (RNGT)

As for the RNGT, each participant in both the Sardinian and LTU samples was tested individually. The researchers asked each participant for their availability to perform a brief test over the phone. It was explained that the test would have been simple and very short. The experimenter also underlined that the test should have been taken in a quiet room, without the presence of other people. Following these explanations, a telephone appointment was set. Subsequently, one of the researchers, called each participant over the phone and read the following instructions either in Italian or in English: “Your task is to produce numbers from 1 to 5 in a random order. To give you an idea of what the task requires, imagine you roll a fair die. Each side of the die is equally likely to be selected with every roll, and each roll is independent of the preceding ones. I would like you to attempt to produce a set of numbers as if you were simulating a fair die. Your sequences will be recorded and analyzed to measure how close you were to simulating a random sequence of numbers. You will hear a series of tones at the rate of 1 per 1 s. Please produce a number in between signals, and continue until told to stop (this will be after 100 responses).”

An example of how to space the responses was also presented to further clarify the procedure. After instructions, the test started and participants kept generating random numbers to the pace of a metronome set to 60 beats per minute. The experimenter recorded the numbers said out loud on paper for a count of 100 numbers. The task was recorded with a recording app with the participants consent.

#### Additional Tests and Qualitative Data

After each tournament game, the players participated in a semi structured interview in which they answered questions about strategies used during the game. Specifically, players have been asked to recall their strategies for number selection, if they were aware of any. Players have been also asked if they can predict the opponent’s behavior. Finally, participants ranked the frequency with which they believe they showed each of the possible five patterns of fingers (1–5).

In November and December 2017 a battery of cognitive tests were administered to all tournament players who were available and willing to take the additional tests (*N* = 5). The tests included the SPM Raven’s Standard Progressive Matrices (Raven, 2008) used to assess players basic non-verbal cognitive abilities, the digit span forward and digit span backward tests (Monaco et al., 2013) used to assess verbal working memory and the Paced Auditory Serial Addition Test (PASAT, Gronwall, 1977; Rao et al., 1989) used to assess speed of information processing and the Wisconsin Card Sorting Test (WCST, Heaton et al., 1993) used to evaluate the ability to use environmental feedback to change cognitive strategies.

As not all the participants were available to take the additional tests, we cannot include the results as a factor in the general analysis. Furthermore, considering the very small sample, they cannot be used to make inferences. We are going to merely discuss the results of the additional tests as Supplementary Information about the features of the population under scrutiny.

#### Coding of the Data

The Morra video recordings were analyzed by two independent observers who, for each game, transcribed the sequences of numbers shown with the hands and spoken by the players. As the sequences of shown numbers vary in each game, we needed to standardize the length of sequences before the analysis. Sequences of randomly generated numbers from Morra games vary in length for two main reasons: different games had different length and the amount of numbers played by each one of the players in a team also varied. Consequently, to be able to analyze the randomness values of the sequences, we needed to standardize the length of all sequences. In order to not exclude too many sequences because of insufficient length, while preserving the robustness of the randomness analyses, we decided to include all sequences with a minimum length of 57 numbers, as a tradeoff between sequence length and number of sequences. For sequences longer than 57, we extracted the first 57 numbers. Consistently, to be able to correctly compare the number sequences from Morra and RNGT, we extracted the first 57 numbers from the RNGT number sequences. All the number sequences from Morra games in Sardinia and at LTU and from RNGTs were analyzed with the software RGCalc (Towse and Neil, 1998) to obtain the following measures of randomness: Redundancy, Frequency of paired responses; NSQ null-score-quotient; coupon score and repetition gap mean. The abovementioned indexes are described below.

#### Randomness Indexes

##### Redundancy (R)

It is calculated as a percentage, with 100% representing maximum redundancy and 0% perfect randomness (see Towse and Neil, 1998, for the calculation of the index). A sequence can be defined redundant, thus less random, inasmuch as parts of it allow to make better-than-chance predictions of subsequent parts.

##### Frequency of paired responses (FPR)

It is an assessment of the distribution of all response pairs in the sequence. Values lie between 0 and 1, FPR rises as particular pair combinations are often repeated. FPR is often described as RNG-index (cf. Towse and Neil, 1998). We prefer FPR to avoid possible confusion of the index with the general RNG task.

##### Null-score quotient (NSQ)

Null-score quotient indicates the percentage of diagrams permutations that do not appear within the subject response set (Guttmann, 1967). The greater the NSQ quotient the worse the performance in terms of randomness.

##### Variability – response cycling

It is a measure of the mean number of responses produced before all the response alternatives are given (Ginsburg and Karpiuk, 1994). The index is also described as coupon by Towse and Neil (1998). In our specific case, as the lowest possible coupon value in Morra is 5, values that get close to five indicate a good performance in terms of variability. The maximum possible value is equal to the number of responses provided (i.e., 57 in our case).

##### Repetition gap mean

It is the average of the number of items produced before an item is repeated. In Morra, with a set-size of 5 possible items, the maximum repetition gap is 5, because it is impossible not to repeat an item after 5 iterations. Consequently, the closest the Repetition gap mean value gets to 5 the better the performance in terms of variability.

#### Additional Measures

The participants in the Morra tournament, in a post-tournament interview, provided a rank indicating the frequency with which they believed to have shown each combination of fingers (1, 2, 3, 4, or 5) during the game. Such subjective rank was compared with the actual frequency with which the numbers were played (actual rank). The distance between the actual and subjective ranks is measured as the difference between the position assigned in the subjective rank and the position in the actual rank (see Table 2 for an example).

**TABLE 2:** Example of subjective frequency ranks, actual frequency ranks with which a hypothetical Morra player, respectively, thought to have used and actually used each one of the five numbers during their games.

	“One”	“Two”	“Three”	“Four”	“Five”	Total distance
Subjective rank	5	4	3	2	1	
Actual rank	1	2	5	4	3	
Distance	4	2	0	2	4	12

We used the subjective-actual rank distance as a measure of the declarative memory of the numbers the players just played in their games. The minimal possible distance is zero, expressing perfect memory of the frequency with which the numbers were played and the maximum possible distance is 12 (as in the example shown in Table 2).

### Data Analysis

Due to the limited sample size, we adopted an exploratory approach to data analysis. The two factors of interest were Task (within-participants, 2 levels: Morra vs. RNGT) and Expertise (between-participants, 2 levels: expert vs. beginners), which generate a 2 × 2 factorial design. The dependent variables were the five indices of randomness. Observations were repeated by participants, therefore they were analyzed using linear mixed-effects models (LMM) with random intercepts for participants. To examine the relevance of the effects using an exploratory approach, we performed model selection. Five alternative models were fitted on each index of randomness: Model 0, including only the intercept; Model 1, including only the main effect of Task; Model 2, including only the main effect of Expertise; Model 3, including both main effects of Task and Expertise; Model 4, including both main effects and the interaction between Task and Expertise. The model selection procedure aimed at determining the best fitting model for index. In addition, we tested the following contrasts: randomness performance in Morra vs. RNGT task within each group; randomness performance in beginners vs. experts for each task.

The data analysis was informed by two important features of our data. First, the dependent variables arguably correlate, as they represent different but related aspects of randomness. Therefore, we adopted a multivariate analytic approach. Second, the dependent variables are bound between two extremes, thus likely violating normality and homoscedasticity. Redundancy, for example, has smaller variance when performance is at close-to-top level (i.e., close to 0%). This can be true of all five dependent variables, as they can all be transformed into percentages between two extremes. The “beta” distribution is perhaps the best fit for all dependent variables with generalized LMM (GLMM), with the “beta” family, being the best modeling choice. However, due to the complexity of the “beta” distribution, and after seeing the variables distributions, we favored the “gamma” family, as it models data skewed toward the bottom (which was our case) in a similar way. In addition, we conditioned the choice of whether to adopt GLMM instead of LMM to the observation of the residuals. Specifically, in a preliminary analysis we performed a Shapiro–Wilk normality test and we observed the asymmetry and kurtosis of the residuals for all dependent variables.

A Bayesian analytic approach was adopted for fitting multivariate LMM and GLMM. To our knowledge, there were no alternatives for performing the same multivariate modeling under the frequentist framework. In addition, the use of Bayesian approach, with the consideration of evidence on its continuum, allowed to mitigate the risks of classical statistical inference with a limited sample size (see below the use of evidence ratio and Akaike-weights). A Bayesian framework has also other advantages, however, including describing the phenomenon at hand in a probabilistic way, placing emphasis on estimating parameters with uncertainty, and facilitating the convergence of complex statistical models (e.g., McElreath, 2016). Nevertheless, for those unfamiliar with the Bayesian approach, we have repeated the same analyses using simpler methods such as separate repeated-measures ANOVAs (they are fully reported in the Supplementary Material – Part 1).

The “brms” package (Bürkner, 2018) of the R software, which fits Bayesian models using the Markov chain Monte Carlo (MCMC) algorithm, was used. For each model, 4 chains each with 5,000 iterations were run. Uninformed default priors were used. The “Rhat” index was used to assess convergence. The Rhat was below 1.01 for all estimated parameters, indicating good convergence.

Evidence was quantified using the Widely Applicable Information Criterion (WAIC; Watanabe, 2010). WAIC is analogous to other popular information criteria used in the frequentist framework, such as AIC and BIC. Based on it, we calculated the Akaike-weights and evidence ratios (Burnham and Anderson, 2004). The former indicates the relative probability of each model being the best within a set of alternatives, given the data, whereas the latter indicates the relative likelihood of a model over another. Appraising evidence using these indices reflects a probabilistic account of the phenomenon at hand, offering a range of degrees of uncertainty (including the possibility of non-conclusive evidence), thus reducing the risks of strong statistical inference with limited samples. Nonetheless, according to the popular interpretations used for the Bayes factor (e.g., Schönbrodt and Wagenmakers, 2018), the following interpretation could be adopted for the evidence ratio (ER): ER > 3 suggests moderate evidence in favor of a model over its competitor (ER < 0.33 suggests moderate evidence against it), and ER > 10 (or ER < 0.10) suggests strong evidence. When comparing models differing in one parameter or effect (e.g., with or without interaction), the ER quantifies evidence in favor or against that effect.

We used the ER also to test the above-mentioned contrasts of interest. We specified H_1_ as “contrast is non-zero,” H_0_ as “contrast is zero.” These contrasts can be examined through model parameters, and they were modeled using prior distributions. Specifically, under H_1_ the uninformed default prior was used (i.e., the parameter is freely estimated based on data), while under H_0_ a very narrow prior distribution centered on zero was imposed (i.e., normal distribution with *M* = 0.000, *SD* = 0.001, practically constraining the parameter to be zero). Therefore, the ER indicates the likelihood of the contrast being equivalent to zero.

Lastly, we calculated effect sizes using a non-parametric way, due to the uncertainty on the data distributions. We chose to use the overlap index, calculated using the “overlapping” package (Pastore and Calcagnì, 2019) of R. This allows to estimate the percentage of overlapping between the distributions of data of two different groups or conditions. At large differences overlapping is close to 0 (0%), at null differences overlapping is close to 1.00 (100%). For interpretation, any overlapping below 0.20 suggests an extremely large difference, equivalent to about Cohen’s *d* > 2.

## Results

The descriptive statistics of all dependent variables in all conditions are reported in Table 3.

**TABLE 3:** Means (and standard deviations) for the five indexes of randomness on the randomly generated sequences in Morra and RNGT in the two groups.

Dependent variable	Task	Group		ER	OV
		Beginners	Experts		
Redundancy	Morra	15.53 (4.43)	3.31 (1.74)	>1,000	0.08
	RNGT	1.59 (1.85)	1.29 (2.27)	0.64	0.23
	*ER*	>1,000	66.69		
	*OV*	0.02	0.07		
FPR	Morra	0.57 (0.03)	0.47 (0.03)	>1,000	0.11
	RNGT	0.46 (0.04)	0.46 (0.03)	0.37	0.70
	*ER*	>1,000	0.41		
	*OV*	0.17	0.71		
NSQ	Morra	37.90 (6.53)	15.82 (5.19)	>1,000	0.12
	RNGT	17.13 (8.88)	16.20 (6.04)	0.29	0.48
	*ER*	>1,000	0.37		
	*OV*	0.22	0.50		
Coupon	Morra	35.01 (12.27)	13.14 (5.63)	121.51	0.23
	RNGT	8.22 (2.86)	8.29 (1.80)	0.47	0.64
	*ER*	>1,000	665.14		
	*OV*	0.07	0.34		
Rep. gap.	Morra	3.99 (0.31)	4.63 (0.21)	>1,000	0.22
	RNGT	4.80 (0.21)	4.84 (0.10)	0.52	0.71
	*ER*	>1,000	3.49		
	*OV*	0.12	0.47		

*Evidence ratios reported in the last column refer to the contrast between the two groups (beginners vs. experts). Evidence ratios reported in rows refer to the contrasts between the two tasks (Morra vs. RNGT) within each group. For the same contrasts, overlapping estimates were also reported. ER, evidence ratio; FPR, frequency of paired responses; NSQ, null-score quotient; RNGT, random number generation task. For Redundancy, FPR, NSQ, and Coupon, higher scores indicate worse randomness performance; for Rep. Gap., lower scores indicate better randomness performance.*

Results obtained using simpler frequentist methods are reported in detail in Supplementary Material – Part 1. They do not contradict any of the conclusions reached below. However, they differ on the interpretation of effects or differences reported as “non-significant.”

**FIGURE 1:**
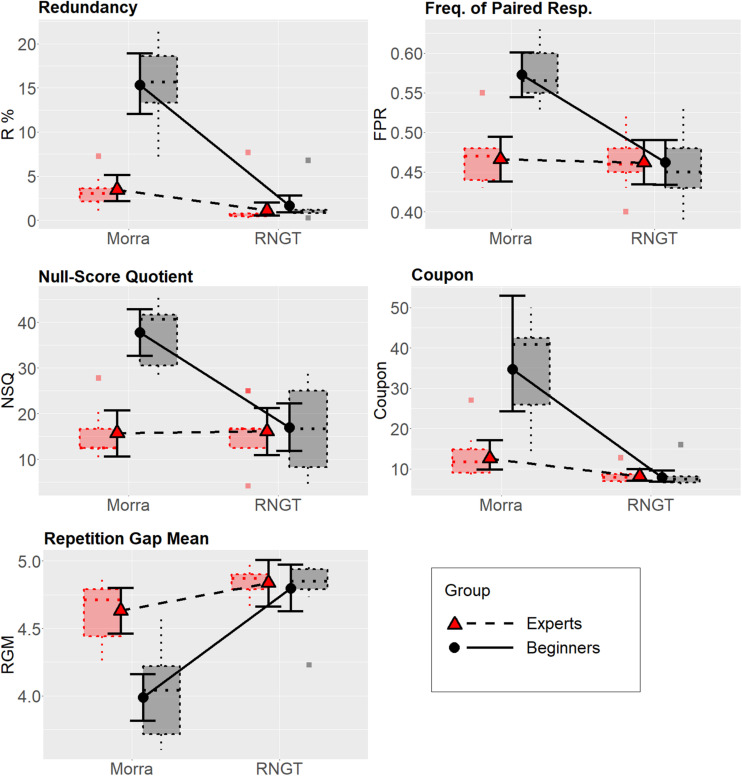
Estimated mean values as a function of the Task × Expertise interaction for each dependent variable. For Redundancy, FPR, NSQ, and Coupon, higher scores indicate worse randomness performance; for Rep. Gap., lower scores indicate better randomness performance. The error bars represent the 95% Bayesian credible intervals calculated with the percentile method from the posterior distributions. Dotted boxplots represent the distributions of the raw data (the central line represents the median; box is the interquartile range; whiskers are the entire range of data; square dots represent outlier observations, i.e., those over 1.5 times the interquartile range beyond the box).

The preliminary analysis on the normality of residuals of the full factor models fitted with LMM suggested that Redundancy and Coupon substantially deviated from the assumptions. At the Shapiro–Wilk test, *W* = 0.89, *p* = 0.002, and *W* = 0.91, *p* = 0.005, respectively (all other *p*s > 0.25); in addition, residuals for both these variables were strongly leptokurtic. Therefore, these two variables were analyzed using GLMM with gamma family (rather than the beta family, which, however, provided practically equivalent results). The remaining three variables had their residuals presenting no strong deviations from normality and were thus analyzed using LMM. Full details can be found in Supplementary Material – Part 2. See also Figure 1 below, for the boxplot showing the distributions of the dependent variables across all conditions.

At the multivariate level, there was very strong evidence in favor of Model 4 (including the Task × Expertise interaction) over any other alternative models. The WAIC-weights indicated over 99.99% probability of Model 4 being the best, given the data. The ER in favor of the model with interaction over the model without it was above 1,000.

At the univariate level, there was strong evidence in favor of Model 4 for each single dependent variable. The WAIC-weights indicated 95.6% probability of Model 4 being the best, given the data, for the Redundancy index (ER = 22.19 in favor of the interaction). For the remaining dependent variables, the probability was always above 99.8% (and all ERs > 100 strongly favored the interactions). All details on WAIC indexes and related probabilities are reported in Supplementary Material – Part 2.

The estimated mean values for all dependent variables as a function of the Task × Expertise interaction, calculated from the posterior distributions of the multivariate GLMM, are shown in Figure 1, along with the boxplot showing data distributions in all conditions.

As regards the contrasts, we investigated them at the multivariate level in the first instance. There was very strong evidence that experts and beginners differed in the Morra condition, ER > 1,000. Conversely, there was also moderate evidence against experts and beginners showing a different performance in randomness in the RNGT condition, ER = 0.02. There was very strong evidence that beginners performed differently in the Morra vs. RNGT condition, ER > 1,000. However, there was also strong evidence that experts differed on the same comparison at the multivariate level, ER > 1,000. Figure 1 shows that the difference in performance between Morra and RNGT was much larger in the beginners than in the experts, however.

Contrasts at the univariate level were also investigated. They showed that, when single variables were considered, some deviations from the multivariate analysis described above emerged. Still, there was very strong evidence of between-group difference in all variables for the Morra task, as well as very strong evidence of difference in performance between the two tasks in the beginners. However, contrary to the multivariate results, experts did not seem to perform differently in Morra vs. RNGT condition with regard to the FPR and NSQ randomness index, and only partially with regard to the repetition gap mean index.

Estimates of overlapping (OV) between the distributions were also reported for the same estimates, as non-parametric effect sizes. The OV measures suggested very large differences between experts and beginner players in all five indices in the Morra condition, with very limited to nearly no overlapping (OVs from about 0.20 to less than 0.10). With regard to the RNGT, on the contrary, there was generally large overlapping between experts and beginners (OVs of about 0.50 or more), with the exception of the Redundancy index (OV = 0.23, which, however, is difficult to interpret because it is very close to ceiling in both groups, practically indicating excellent performance in nearly all participants). Concerning the within-group comparisons, there was very limited to nearly no overlapping in the distributions of the beginner players across the two conditions (Morra vs. RNGT), with OVs ranging from about 0.20 to nearly zero in all randomness indices, suggesting that each beginner perform nearly systematically worse in Morra than in RNGT in terms of randomness. On the contrary, the same OVs ranged around 0.50 in the experts, indicating much more overlapped performances in this group, the only notable exception being Redundancy, in which experts performed clearly worse in Morra than in RNGT (albeit still much better than beginners).

Lastly, we calculated the correlations among the residuals of the final multivariate model (Model 4) for the five dependent variables. Correlations were mostly moderate to strong, and ranged from 0.08 to 0.90, with average being 0.39 (in absolute values). This confirmed the appropriateness of focusing on an analysis at the multivariate model.

### Additional Measures

Participants at the Bitti’s tournaments were interviewed at the end of the tournament. The interviews were transcribed and a qualitative analysis was performed. A complete report of the analysis is included in another article in preparation. In their comments, players show little awareness of the processes involved in the game as they were vague in verbalizing their strategies and the strategies of their opponent. Each participant was also asked the following questions: “What is the number that you played the most during the game? And the second most frequent? And the third? And the fourth? And the least frequent?” These data were used to produce a subjective rank of the frequency with which each player thought he had shown each one of the possible combinations of fingers from “one” to “five.” We compared such subjective rankings with the actual frequency of use of each number. Results are summarized in Table 4.

**TABLE 4:** Comparison between subjective and actual rankings of the frequency of occurrence of hand-numbers in 11 sequences extracted from the games of the Bitti’s tournament.

		First	Sec.	Third	Fourth	Fifth	Dist.-1st	Dist.-2nd	Dist.-3rd	Dist.-4th	Dist.-5th	Tot. dist.	% dist.
Seq. 1	Subjective	4	3	5	2	1	2	2	2	3	3	12	100%
	Actual	2	1	4	3	5							
Seq. 2	Subjective	2	4	3	5	1	1	2	2	1	2	8	67%
	Actual	3	2	1	4	5							
Seq. 3	Subjective	1	5	2	3	4	4	3	0	1	4	12	100%
	Actual	4	1	2	5	3							
Seq. 4	Subjective	3	4	2	5	1	2	2	2	2	4	12	100%
	Actual	1	5	3	4	2							
Seq. 5	Subjective	1	4	3	2	5	1	1	0	0	0	2	17%
	Actual	4	1	3	2	5							
Seq. 6	Subjective	4	2	1	3	5	4	0	2	0	4	10	83%
	Actual	5	2	4	3	1							
Seq. 7	Subjective	4	3	1	5	2	2	2	2	3	3	12	100%
	Actual	5	2	4	3	1							
Seq. 8	Subjective	3	1	5	4	2	3	3	1	1	4	12	100%
	Actual	2	5	4	3	1							
Seq. 9	Subjective	3	4	2	5	1	4	1	2	0	3	10	83%
	Actual	2	1	4	5	3							
Seq. 10	Subjective	4	3	2	1	5	3	1	1	3	0	8	67%
	Actual	3	2	1	4	5							
Seq. 11	Subjective	2	3	1	5	4	1	1	2	1	1	6	50%
	Actual	1	2	3	4	5							

*Maximum score (12) indicates maximum discrepancy (100% distance) between the subjective impression of the occurrence of hand-numbers and their actual frequency.*

As seen in Table 4, the level of matching between the perceived and the actual frequency of the played combinations is very low. The average distance between perceived and actual frequency is 9.45 in a scale ranging from 0 to 12. As shown in Table 4, it is remarkable that in five sequences out of 11 the discrepancy between the actual and the subjective rankings was the highest possible, indicating that, in spite of their expertise, the players were not at all aware of the frequency with which they showed numbers during their games. We argue that the very low correspondence between subjective and actual rankings of the frequency of the occurrence of numbers indicates a very low level of awareness of the defensive strategies in Morra.

Finally, some of the tournament participants accepted to participate in a battery of neuropsychological tests (see Table 5) including the Raven’s progressive matrices, Span (forward and backward) the Pasat (3” and 2”) and the Wisconsin sorting card test (WCST).

**TABLE 5:** Average scores of expert Morra players and reference scores in the cognitive tests battery.

Participant	RAVEN’s (total score)	Span forward	Span backward	Wisconsin sorting card test (errors)	PASAT 2″
#1	17	5	3	84	17
#2	40	6	3	27	30
#3	29	6	5	70	44
#4	30	8	6	52	60
#5	32	5	6	43	46
Mean	29.6 (8.3)	6 (1.2)	4.6 (1.5)	55.2 (22.4)	39.4 (16.4)
Test norm mean	44.94	6	5	25.6	35.1

The five players have shown a Raven SPM test score of 29.6, which is below the 25th percentile. The forward and backward span indicate an average verbal working memory performance (*M* = 6 and *M* = 4.6, respectively) within the norm of the test (Monaco et al., 2013). The 2-s PASAT performance is below the 5th percentile for 2 players out of 5, while the other 3 are within the norm of the test (Rao et al., 1989). Finally, players also have a low average performance in the WCST (*M* = 55.2), which is below the 30th percentile. These results are surely incomplete and not necessarily representative of the whole sample, but seem to preliminary suggest that the superior random number generation ability in Morra shown by experts are not associated with generalized superior cognitive functioning measured through standard cognitive tests. Further systematic tests should be conducted to investigate this hypothesis.

## Discussion

In this study we wanted to investigate whether it is possible to proceduralize the generation of random sequences of numbers. In order to do so, we compared randomness coefficients of number sequences produced by experts and non-experts players of Morra during RNGT and during Morra games. We hypothesized that expert players would outperform beginners in terms of randomness in Morra, but not in RNGT. We also expected naïve players to show largely worse randomness scores in Morra than in RNGT, whereas the same scores were expected to be similar in expert players.

Results indicated that the measures of redundancy of responses (*R*), *of f*requency of paired responses (*FPR*), of percentage of missing diagrams permutation (*NSQ*), of response cycling and of average repetition gap were all largely affected by expertise, with beginners performing poorer than experts. Most importantly for our hypothesis, in all measures, experts largely outperformed beginners in terms of randomness while playing Morra, while the two groups were almost perfectly equivalent while performing RNGT. In fact, even expert players showed slightly superior randomness in Morra than in RNGT in two randomness indices (R and response cycling). However, these differences were very small in absolute terms, and largely smaller than those observed in the beginners.

All the additional tasks that experts performed while playing Morra, (motor acts, sum the two hands, selection of spoken numbers and many other processes) had very limited or even no impact, depending on the randomness index considered, on their ability to select the numbers randomly. We conclude that Morra experts were largely able to proceduralize random number generation during Morra. Interestingly, expert Morra players were not different from beginners when they perform RNGT in isolation, without the interference of additional tasks. This finding suggests that Morra experts do not always outperform novices in random sequence generation, but that they are specifically better only when random sequence generation is performed in the presence of concurrent tasks. It should also be noted that both groups performed excellently in RNGT, however, with randomness performance close to ceiling in some variables.

These results have theoretical implications within the debate about automatic versus voluntary processing in random sequence generation. In fact, while previous studies supported the hypothesis that producing random or unpredictable sequences requires a substantial attentional component (see for example Baddeley, 1966), our results suggest that producing random sequences, specifically numerical ones, can be relatively free from attentional costs in trained individuals. More specifically, the ability by expert players to produce excellent levels of randomness during both Morra and RNGT, and even perfectly comparable across the two conditions in some aspects, suggests that expertise can drastically reduce the cost of the concurrent tasks required to play Morra on the level of randomness of the generated hand-numbers sequences. This result is in contrast with previous findings (Towse and Valentine, 1997; Baddeley et al., 1998; Towse and Cheshire, 2007) who found that concurrent tasks were able to disrupt performance in Random Sequence Generation Tasks. Random number generation, is often described as a quintessentially sequential, slow, and attentive process. Such kind of processes, in the dual process theory is assumed to be performed by the so-called system 2 (Stanovich and West, 2000; Kahneman, 2011), which is controlled, requires high effort, is mostly voluntary, requires attention, is slow, and suffers from interference. However, when performed by expert players in Morra games, random number generation does seem to suffer from the contemporary involvement of the cognitive system in the concurrent operations necessary to play the game. The impenetrability to interference from concurrent tasks is often considered as an evidence of automaticity (see the seminal work of and Shiffrin and Schneider, 1977 as an example) of tasks which do not require attention. As RNGT has been used as an index of attention deployment and controlled processing (Evans, 1978; Evans and Graham, 1980; Jahanshahi et al., 2006) it seems important to point out that the randomness indexes in Morra do not suffer from interference. Such result support the idea that even processes that are considered exemplary instances of controlled and voluntary cognitive tasks are susceptible to certain degrees of automatization. Previous evidence of a lack of interference between an overlearned task and RNGT has been provided by Evans and Graham (1980). They asked participants to simultaneous perform a two-hand coordination task and RNGT. They found that, after a marked deterioration during the initial skill acquisition phase followed by a progressive improvement, participants completely recovered their original baseline levels of randomness during the overlearning trials. This finding is particularly relevant because, like in Morra, Evans and Grahams task involved both hand coordination and overlearning. In Morra, the motor skills can even be more central in the process of automatization. In fact, in Evans and Grahams the hand coordination task and RNGT are different, mutually interfering tasks that still can be performed at baseline levels after overlearning. In Morra, where the arm-hand movement is inextricably associated with number generation, it is likely that the motor task can be performed rather automatically from early stages of learning. Additional support of the automatization of random number generation strategies comes from the analysis of the after-game surveys. Specifically, the quantitative analysis of the distance between the ranking of the frequency with which they actually played each number and their subjective impression of it indicates that players did not have conscious recollection of their own defensive strategies.

How is it possible that Morra players, in contrast with results from previous studies on random sequence generation, pay minimized attentional costs when the task is associated with concurrent tasks? We tend to exclude that superior randomness shown by expert players is a consequence of a more efficient general cognitive functioning. In fact, while our data are partial and not necessarily representative of the whole sample, a battery of cognitive tests preliminary suggest that expert Morra players do not score higher than the normative averages in several cognitive tests measuring memory, attention, executive function and fluid intelligence. As the Morra expert all had hundreds, and possibly thousands of hours of training in random number generation during Morra, we tend to think that expertise can be a crucial factor. We argue that, if enough training time is provided, random sequence generation can be proceduralized and become relatively effortless, unconscious, fast and immune from interference of concurrent tasks. An alternative account for the superior randomness indexes of experts in Morra is familiarity with the task. However, if greater randomness would be caused by mere familiarity and not by proceduralization in the presence of concurrent tasks, we would expect to find a better performance of experts in Morra, which is a very familiar task for the experts than in RNGT, which is a completely new task to them. Result suggest that this is not the case. Actually, for some of the randomness indexes, we found a slightly superior performance of experts in RNGT than in Morra. It should be noted that random number generation in Morra and RNGT are not fully comparable. Specifically, random sequence generation in Morra is not an independent process isolated by other concurrent tasks like in experimental paradigms used in previous studies (Towse and Valentine, 1997; Baddeley et al., 1998; Towse and Cheshire, 2007). As in Morra random number generation likely interacts with other strategies and processes, the comparability with random generation tasks traditionally used in psychological research, and in this study, is limited. In fact, in Morra, all processes are linked to each other. In particular, when numbers are simultaneously shown and verbally expressed, the spoken and shown numbers are inextricably associated. Specifically, for a spoken number to be successful, the player will say a number which is the sum of the number shown with the hand and the number he/she predicts to-be-shown by the opponent player. Also, and consequently, the spoken number should always be greater than the shown number. Finally, the difference between the spoken number and the shown number should always be equal or lesser than 5. Morra is better defined as a binding process between tasks instead as a competition between concurrent tasks. Such binding requires a long and sustained training and results in expert performances able to produce number sequences that are rapid, not entirely conscious and, most importantly, that are characterized by high level of randomness. As an influence of motor control and body posture in RNGT has been already found (Loetscher et al., 2008), it would be interesting to consider the possibility that instead of detrimental, the concurrent processes could rather facilitate random number generation in Morra. Following this line of reasoning, a follow up experiment could verify whether Morra experts would show enhanced levels of randomness in RNGT if, instead of verbalizing numbers they would use show them with theirs hands, like in a Morra game. In fact, the modality of number expression (hand in Morra vs. voice in RNGT) could be a reason why the excellent randomization skills of Morra experts do not seem to transfer to other settings of random sequence generation like RNGT.

In this first cognitive analysis of the Morra game we considered random number generation in isolation in a semi-naturalistic setting. Further studies should consider how the generation of spoken numbers (attack strategies) and hand number generation interact. We recognize that when a multi-componential task is under scrutiny, it is complicated to disentangle the different components and understand their singular contributions to the whole process. However, we believe that experimental manipulations of conditions in the laboratory could provide creative ways to analyze the separate contribution of the different components in the multitasking process. The comparison between different modality of number expression in random number generation (verbal vs. hand-gesture) we mentioned earlier in the discussion is an example. Also, playing against a computer that generates the number in different modalities and formats can help isolate the specific contributions of integrated processes. Likewise, the use of techniques for the automatic acquisition of Morra sequences could help to reduce the time necessary for data acquisition and tabulation and allow testing a larger and more balanced sample of participants.

Finally, Morra could be used as an original paradigm to study a number of different mechanisms and processes like, to mention just a few, probability estimation in implicit learning, memory for sequences, socio-affective components in competitive games and the effectiveness of culturally specific pedagogical approaches in the development of mathematical skills.

## Data Availability Statement

The raw data supporting the conclusions of this article will be made available by the authors, without undue reservation, to any qualified researcher.

## Ethics Statement

The studies involving human participants were reviewed and approved by the IRB Committee – Lawrence Technological University. The patients/participants provided their written informed consent to participate in this study.

## Author Contributions

FD conceived of the original idea and theoretical framework. FD, CM, and RF conceived and planned the experiments. FD, MB, and CM carried out the experiments. AZ conducted the numerical simulations. ET conducted the statistical analysis. FD and MB wrote the main manuscript with the support, contribution, and inputs of all the authors. All authors contributed to the final version of the manuscript.

## Conflict of Interest

The authors declare that the research was conducted in the absence of any commercial or financial relationships that could be construed as a potential conflict of interest.
