# Supplementary material for: The Morra Game as a Naturalistic Test Bed for Investigating Automatic and Voluntary Processes in Random Sequence Generation
Source: Front Psychol. 2020 Sep 24;11:551126. doi: 10.3389/fpsyg.2020.551126 (PMC7541701; doi:10.3389/fpsyg.2020.551126)
Supplement: Supplementary file 1 [file Table_1.DOCX]

**Supplemental Material**

**The Morra game as a naturalistic test bed for investigating automatic and**

**voluntary processes in random sequence generation**

***Summary of the content:***

**PART 1 – Frequentist analysis: Results obtained with repeated-measures ANOVAs**

**PART 2 – Additional information on manuscript Results**

**References (supplemental)**

**PART 1 – Frequentist analysis: Results obtained with repeated-measures ANOVAs**

For the readers unfamiliar with Bayesian methods of estimation, we conducted an additional set of analyses adopting frequentist methods and the traditional assumptions for linear models. These include homoscedasticity and normal distribution of the residuals. Due to the difficulty to fit repeated-measures multivariate analysis with frequentist methods, the multivariate indexes were not calculated.

A series of factorial repeated-measures analyses of variance (ANOVAs) were performed to assess the influence of the task and expertise on the five indexes of randomness in number random generation. The 2 X 2 design included Task (Morra vs RNGT) as a within-participants factor and Expertise (experts vs beginners) as a between-participants factor.

Table S1 below reports the descriptive statistics and it summarizes the results of post-hoc analyses from the ANOVAs on the five indexes of randomness.

As regards the redundancy of responses (R), Expertise significantly affected R, *F*(1,16) = 32.83, *p* < .0001, *η*^2^ = .67, indicating that the beginners showed significantly higher levels of redundancy (less variability) than experts. Task was also significant, showing that the RNG generated less redundancy than Morra, *F*(1,16) = 83.74, *p* < .0001, *η*^2^ = .84. The interaction between expertise and task was also significant, *F*(1,16) = 46.81, *p* <.0001, *η*^2^ = .75. Post-hoc analysis indicated that while beginners were less redundant in their RNG than in Morra (*p* < .0001), experts did not differ while performing either Morra or RNGT (*p* = .122). Also, experts and non-experts where equivalent while performing RNG (*p* = .83), while experts were significantly less redundant than beginners when performing Morra (*p* < .0001).

As regards the frequency of paired responses (*FPR*), Expertise significantly affected FPR, *F*(1,16) = 17.22, *p* <.001, *η*^2^ = .52, with beginners showing significantly higher levels of FPR than experts. *Task* was also significant, indicating that the FPR generated less predictable set of pairs (digrams) than Morra, *F*(1,16) = 19.03, *p <* .001, *η*^2^ = .54 . The interaction between Expertise and Task was also significant, *F*(1,16) = 16.20, *p* < .001, *η*^2^ = .50. Post-hoc analysis indicated that while beginners performed better in the RNGT than in Morra in terms of predictability of their digrams (*p* < .0001), experts did not differ while performing either Morra or RNGT (*p* = .81). Also, experts and non-experts where equivalent while performing FPR (*p* = 1), while experts were significantly less predictable than beginners when performing Morra (*p* < .0001).

As regards the percentage of missing diagram permutations expressed by the null-score quotient (NSQ), Expertise significantly affected NSQ, *F*(1,16) = 27.54, *p* <.0001, *η*^2^ = .63, indicating that the beginners showed a significantly higher number of missing digrams than experts. Task was also significant, showing that participants missed more digram permutations in the Morra task than in the RNGT, *F*(1,16) = 15.41, *p* < .001, *η*^2^ = .49. The interaction between Expertise and Task was also significant, *F*(1,16) = 16.57, *p* <.001, *η*^2^ = .51. Post-hoc analysis indicated that while beginners missed less permutations during the RNG than in Morra (*p* < .0001), experts did not differ while performing either Morra or RNGT (*p* = .919). Also, experts and non-experts where equivalent while performing RNG (*p* = .79), while experts missed less combinations than beginners when performing Morra (*p* < .0001).

As regards response cycling, Expertise significantly affected the response cycling score, *F*(1,16) = 16.82, *p* < .001, *η*^2^ = .51, indicating that the beginners needed a higher number of trials to provide all possible responses. Task was also significant, showing that cycling in RNGT needed less trials than in Morra, *F*(1,16) = 49.61, *p* < .0001, *η*^2^ = .76. The interaction between Expertise and Task was also significant, *F*(1,16) = 23.86, *p* < .001, *η*^2^ = .60. Post-hoc analysis showed that while beginners had shorter cycling in RNGT than in Morra (*p* < .0001), experts did not differ while performing RNG or Morra (*p* = .146). Also, experts and non-experts were equivalent while performing RNGT (*p* = .98), while experts had significantly lower response cycling scores than beginners when performing Morra (*p* < .0001).

As regarding the repetition gap mean between identical responses, Expertise significantly affected the repetition gap, *F*(1,16) = 17.44, *p* <.001, *η*^2^ = .52, indicating that the beginners repeated identical numbers after a smaller number of responses than experts. Task was also significant, showing that the RNG generated bigger gaps than Morra, *F*(1,16) = 44.16, *p* < .0001, *η*^2^ = .73. The interaction between Expertise and Task was also significant, *F*(1,16) = 15.56, *p* <.001, *η*^2^ = .49. Post-hoc analysis indicated that while beginners showed shorted repetition gaps in RNGT than in Morra (*p* < .0001), experts did not differ while performing either Morra or RNGT (*p* = .074). Also, experts and non-experts where equivalent while performing RNGT (*p* = .73), while experts had longer gaps than beginners when performing Morra (*p* < .0001)

**Table S1**. Means (and standard deviations) for the five indexes of randomness on the randomly generated sequences in Morra and RNGT in the two groups. Significance levels reported in the last column refer to the post-hoc comparisons between the two groups (Beginners vs Experts). Significance levels reported in rows refer to the post-hoc comparisons between the two tasks (Morra vs RNGT) within each group.

| *Dependent variable* | Task | Group | |  |
| --- | --- | --- | --- | --- |
|  |  | Beginners | Experts | *p-value* |
| Redundancy | Morra | 15.53 (4.43) | 3.31 (1.74) | ** |
|  | RNGT | 1.59 (1.85) | 1.29 (2.27) | *ns* |
|  | *p-value* | ** | *ns* |  |
| FPR | Morra | .57 (.03) | .47 (.03) | ** |
|  | RNGT | .46 (.04) | .46 (.03) | *ns* |
|  | *p-value* | ** | *ns* |  |
| NSQ | Morra | 37.90 (6.53) | 15.82 (5.19 | ** |
|  | RNGT | 17.13 (8.88) | 16.20 (6.04) | *ns* |
|  | *p-value* | ** | *ns* |  |
| Coupon | Morra | 35.01 (12.27) | 13.14 (5.63) | ** |
|  | RNGT | 8.22 (2.86) | 8.29 (1.80) | *ns* |
|  | *p-value* | ** | *ns* |  |
| Rep. gap. | Morra | 3.99 (.31) | 4.63 (.21) | ** |
|  | RNGT | 4.80 (.21) | 4.84 (.10) | *ns* |
|  | *p-value* | ** | *ns* |  |

* *p* < .05; ** *p* < .001 ns = *p* > .05

*Note*. FPR = frequency of paired responses; NSQ = null-score quotient; RNGT = random number generation task.

**PART 2 – Additional information on manuscript Results**

*Preliminary analysis on the normality of residuals*

A first analysis was conducted on the normality of residuals extracted from the full factor linear mixed-effects model (fitted with maximum likelihood), including the Task by Expertise interaction, on all 5 dependent variables. The models were fitted using the “lme4” package (Bates, Maechler, Bolker, & Walker, 2015) of the R software. Asymmetry (skewness) and kurtosis of the residuals were calculated with the “moments” package (Komsta & Novomstky, 2015) of R. As normative reference, for the Gaussian distribution, skewness = 0, kurtosis = 3. We considered any deviation of less than .5 in skewness and less than 1.0 in kurtosis as mild. See details in Table S2. Figure S1 shows the residuals distributions in boxplots.

**Table S2**. Results for the Shapiro-Wilk normality test, skewness and kurtosis, for the residuals of all dependent variables.

| *Dependent variable* | Shapiro-Wilk normality test | | Skewness | Kurtosis |
| --- | --- | --- | --- | --- |
|  | *W* | *p-value* |  |  |
| Redundancy | .89 | .002 | .06 | 4.87 |
| FPR | .97 | .38 | .42 | 2.68 |
| NSQ | .97 | .36 | .05 | 2.18 |
| Coupon | .91 | .005 | -.81 | 5.16 |
| Rep. gap. | .96 | .26 | -.18 | 3.63 |


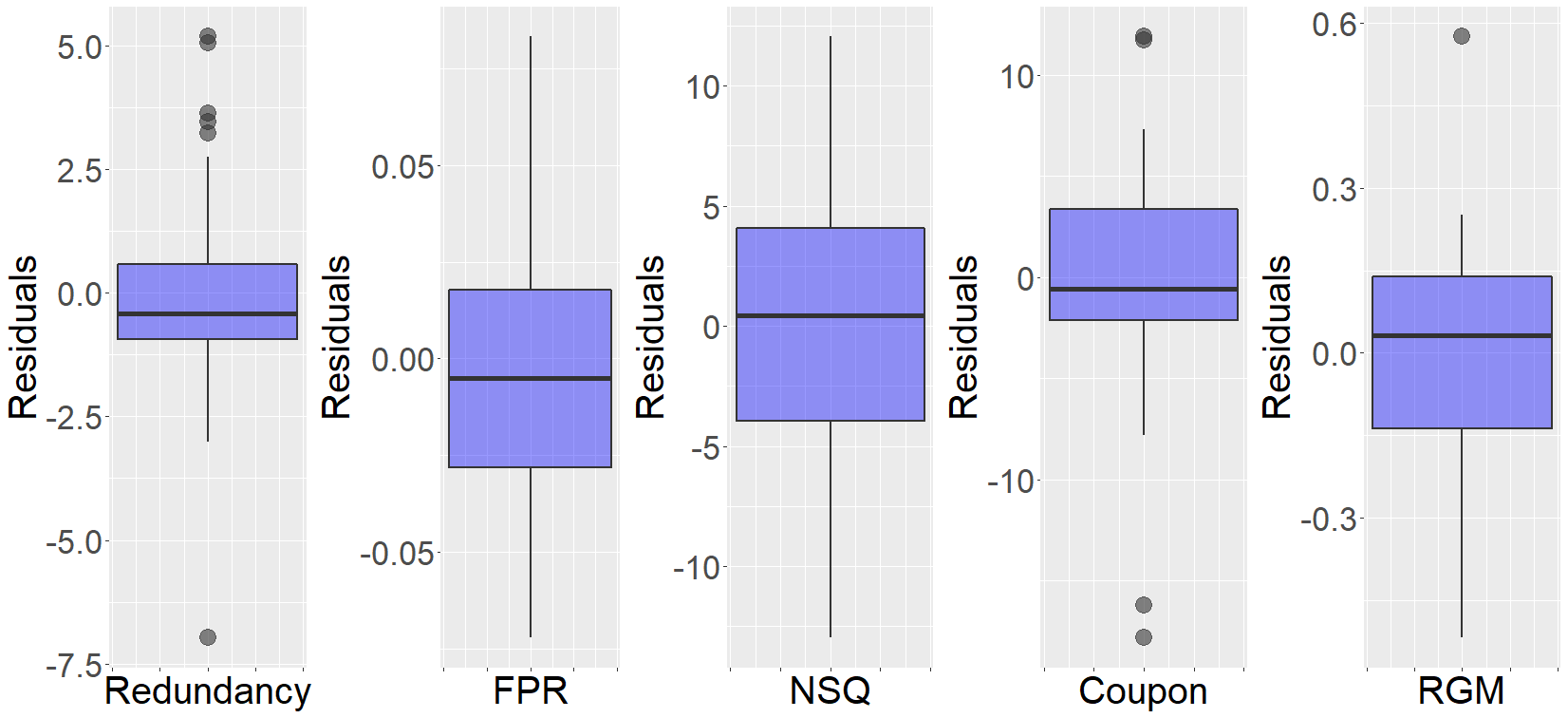


**Figure S1**. Boxplots showing the distributions of residuals of all dependent variables. The thick line in the middle shows the median. The box represents the interquartile range. Whiskers represent the entire range. Outliers (i.e., those 1.5 times the interquartile range beyond the box) are shown as circular dots. FPR = frequency of paired responses; NSQ = null-score quotient; RGM = repetition gap mean.

*Additional tables*

**Table S3**. WAIC and WAIC-weights of the five alternative competing models for all five dependent variables.

| *Dependent variable* |  | Model 0 | Model 1 | Model 2 | Model 3 | Model 4 |
| --- | --- | --- | --- | --- | --- | --- |
|  |  | (Intercept) | Task | *Expertise* | Task + Expertise | Task X Expertise |
| Redundancy | WAIC | -136.6 | -158.8 | -138.8 | -179.0 | -185.2 |
|  | WAIC-weights | 0% | 0% | 0% | 4.4% | 95.6% |
| FPR | WAIC | -95.1 | 103.3 | -110.1 | -110.9 | -124.5 |
|  | WAIC-weights | 0% | 0% | 0% | 0.1% | 99.9% |
| NSQ | WAIC | 283.6 | 277.0 | 276.1 | 266.9 | 251.7 |
|  | WAIC-weights | 0% | 0% | 0% | 0% | 99.9% |
| Coupon | WAIC | -33.5 | -66.9 | -41.7 | -70.2 | -86.1 |
|  | WAIC-weights | 0% | 0% | 0% | 0% | 100% |
| Rep. gap. | WAIC | 43.5 | 26.6 | 38.8 | 17.4 | 5.4 |
|  | WAIC-weights | 0% | 0% | 0% | 0.2% | 99.8% |

**Table S4**. Bivariate correlations among the residuals of the final multivariate model (Model 4) for the five dependent variables.

|  | 1. | 2. | 3. | 4. | 5. |
| --- | --- | --- | --- | --- | --- |
| 1. Redundancy | - |  |  |  |  |
| 2. FPR | .46 | - |  |  |  |
| 3. NSQ | .52 | .90 | - |  |  |
| 4. Coupon | .51 | .13 | .26 | - |  |
| 5. Rep. gap. | -.69 | -.31 | -.36 | -.68 | - |

**References (supplemental)**

Bates, D., Maechler, M., Bolker, B., & Walker, S. (2015). Fitting Linear Mixed-Effects Models Using lme4. *Journal of Statistical Software, 67*, 1-48. doi:10.18637/jss.v067.i01

Komsta, L., & Novomestky, F. (2015). *moments: Moments, cumulants, skewness, kurtosis and related tests*. R package version 0.14. URL:https://CRAN.R-project.org/package=moments
